# Permanent crop cover as a strategy for drought-resistant viticulture: insights on how rhizosphere metagenomics influences leaf-level -omics for an enhanced overall plant response

**DOI:** 10.3389/fpls.2025.1543171

**Published:** 2025-05-29

**Authors:** Iván Jáuregui, María Ancín, José M. García-Mina, Angel M. Zamarreño, Ariadna Iglesias-Sanchez, Igor Florez-Sarasa, Iker Aranjuelo

**Affiliations:** ^1^ Department of Sciences, Public University of Navarra (UPNA), Pamplona, Spain; ^2^ AgroBiotechnology Institute (IdAB), Consejo Superior de Investigaciones Científicas (CSIC)-Government of Navarre, Mutilva, Spain; ^3^ Universidad de Navarra, Instituto de Biodiversidad y Medioambiente BIOMA, Pamplona, Spain; ^4^ Universidad de Navarra, Facultad de Ciencias, Departamento de Biología Ambiental, Pamplona, Spain; ^5^ Centre for Research in Agricultural Genomics (CRAG), Barcelona, Spain; ^6^ Institut de Recerca i Tecnología Agroalimentàries (IRTA), Barcelona, Spain

**Keywords:** vineyard, crop cover, drought, metabolomics, transcriptomics, metagenomics, WUE

## Abstract

The viticulture sector is currently threatened by climate change, impacting grape quality and yield through altered weather patterns and reduced soil health. The incorporation of cover crops can significantly bolster sustainability by enhancing soil moisture retention and structural integrity, both of which are essential for the enduring viability of vineyards in the long term. Cover cropping presents numerous advantages, such as the enhancement of soil health, mitigation of erosion, and facilitation of nutrient cycling; however, it may also pose certain short-term risks, including competition for vital resources like water. In spite of the progress made in comprehending the advantages of cover crops in vineyard settings, the intricate dynamics between plant–microbe interactions and the leaf-level metabolic responses of grapevines at the leaf level to drought conditions remain unexplored. This study examines the impact of water availability and green cover (comprising perennial ryegrass and *Trifolium repens*) on grapevine photosynthetic and metabolism efficiency, positing that crop cover fosters a microhabitat that bolsters microbial communities and drought resilience. Through comprehensive examinations of gas exchange, isotopic analysis, metabolomics, transcriptomics, and soil metagenomics, this study clarifies the relationships among irrigation methodologies, photosynthesis, and soil health, ultimately aiding in the fortification of agricultural resilience in the face of climate change. Our investigation demonstrates that the adoption of cover crops yields unexpected immediate benefits in bolstering drought resilience for vineyards. Despite an observed increase in overall evapotranspiration during drought conditions, the use of cover crops facilitated carbon accumulation and enhanced osmolyte-acting metabolites (including sugars and sugar alcohols) and abscisic acid (ABA) concentrations, alongside a comprehensive molecular adaptation to drought stress. Moreover, cover cropping was shown to promote the expression of defense-related pathways, while vineyards devoid of cover crops exhibited minimal transcriptional responses; certain taxa exhibited responses contingent upon the treatment, with Tistrellales and Gaiellales being linked to crop cover under favorable conditions, whereas *Rhizoctonia* demonstrated a strong association with rhizospheric soil during drought conditions when crop cover was present. Our study is the first to show that cover cropping can boost cash crop resilience to drought through intricate plant–soil–microbe interactions, providing benefits from the outset.

## Introduction

The cultivation of grapevines is currently facing significant challenges due to climate change. This phenomenon threatens the quality and yield of grapes by altering temperature and precipitation patterns, as well as leading to the depletion of organic matter in the soil. Given that grapevines are perennial crops, an adaptation strategy by changing varieties presents considerable difficulties (Van Leeuwen et al., 2019). Integrating crop covers into these systems enhances sustainability by playing a significant role in the conservation of soil moisture and enhancement of soil structure (Buesa et al., 2021) with the goal of maintaining vineyard productivity and ensuring the long-term viability of the wine industry.

Cover cropping presents a multitude of advantages that can enhance both productivity and sustainability (Novara et al., 2021). First, the utilization of cover crops enhances the soil’s composition through the increase of organic material and the promotion of soil aggregation (Steenwerth and Belina, 2008), improving water infiltration and retention. Furthermore, this cropping system aids in the prevention of soil erosion by providing a protective ground cover against wind and water damage. Second, they can contribute to reproductive growth management (Muscas et al., 2017). Third, green cover can contribute significantly to nutrient cycling, with legumes, for example, being able to fix atmospheric nitrogen (N) (Abad et al., 2021b) while having some effect on inhibiting pests (Abad et al., 2021a). Fourth, cover crops foster beneficial soil microorganisms, heightening soil fertility and plant well-being (Baumgartner et al., 2005) while reducing pest appearance (Rosa et al., 2022). Nevertheless, while green cover crops provide numerous advantages for vineyards, they also pose potential obstacles. There is a risk that green cover may disrupt maintenance procedures on the farm. Furthermore, cover crops may serve as habitats for pests and diseases that could potentially harm the grapevines or that grass can compete with N (Abad et al., 2021b). A notable concern about cover cropping is the competition for water and nutrients between the cover crops and grapevines, particularly in areas with limited water resources (Romero et al., 2022). Therefore, addressing this competition necessitates meticulous planning and supervision, leading to heightened labor and management expenses. Despite these challenges, there is a general agreement that incorporating green cover in perennial crops elevates overall sustainability, resulting in more robust agricultural systems and healthier ecosystems.

Global climate models suggest that Europe will face reduced water precipitation and increased temperatures in the upcoming years. This prediction raises alarms about the future of plant growth and agricultural productivity amid changing environmental conditions. When evaluating plant photosynthetic capacity, and consequently plant growth, one must consider that CO_2_ assimilation is heavily influenced by environmental factors and agricultural practices and that there is a significant bias between leaf and canopy photosynthesis (Niinemets and Anten, 2009). Research conducted at both leaf and whole plant levels indicates that drought can lead to substantial reductions in photosynthesis and plant growth, exceeding 80% (Lawlor and Cornic, 2002). These inhibitory effects are linked to stomatal closure to minimize water loss, oxidative stress harm, and reduced activity of genes related to photosynthetic processes. While leaf-level measurements provide detailed insights into stomatal activity and photosynthesis, they often fail to capture the spatial and temporal variabilities inherent in the canopy (Soba et al., 2020). Canopy-level assessments, in contrast, integrate the cumulative effects of environmental factors such as light intensity, temperature, and vapor pressure deficit, which vary across canopy layers. These factors can significantly influence stomatal behavior and, consequently, gas exchange and water loss. In this sense, the stable isotope of carbon, with a nucleus containing six protons and seven neutrons, named carbon-13 (^13^C) isotope, has been widely identified as a reliable marker characterizing plant responses to drought stress over time by allowing for a more integrated evaluation of carbon assimilation and water use efficiency compared to conventional measurement techniques (Farquhar and Richards, 1984; Bchir et al., 2016). Surprisingly, integrative approaches using different techniques for capturing drought impact on vineyards with crop cover are rarely found in the literature.

Cutting-edge methodologies such as metagenomics are shedding light on the complexity of the interaction between plants and their immediate microbial communities in the soil-rhizosphere communities, unveiling their significance in improving soil quality and resilience (French et al., 2021). These approaches bring light to plant mechanisms for alleviating drought stress via the modulation of root-associated microbial consortia (Xu et al., 2018). Root-associated microorganisms significantly enhance plant drought resilience through various mechanisms, such as the synthesis of osmolytes and exopolysaccharides that improve soil water retention (Schmidt et al., 2024). Furthermore, recent in-depth research has shown that both annual and perennial cover crops have a positive but variable effect on soil health by promoting a wide range of microbial communities (Beillouin et al., 2021). Specifically for vineyards, Vink et al. (2021) found that soil bacterial communities in grapevines are shaped by both plant association and the environment, which in turn may define the health and growth of the grapevines. Additionally, a vast diversity of fungi was found in the soil of cover crops planted between the rows (Hendgen et al., 2018). Even in the short term, cover crops can significantly improve soil quality (Cardinale et al., 2022). Despite the significant progress in understanding the use of cover crops in vineyards for sustainable viticulture, there is still limited understanding regarding the relationship between plant–rhizosphere community interaction and environmental stressors like edaphic drought.

The current research delves into the effects of water availability and the application of green cover based on perennial ryegrass and *Trifolium repens* on the photosynthetic efficiency and carbon metabolism of grapevine plants. Our working hypothesis is that crop cover provides an extra microhabitat for microbial communities, enhancing the resilience to drought conditions of the holobiont comprised of soil, crop cover, and the vineyard. Through extensive gas exchange analyses at leaf and canopy levels, the study investigates how young grapevines respond to varying irrigation levels (full versus 50% capacity) in terms of photosynthesis and transpiration rates. Carbon isotope composition, metabolite, and hormone profiling were examined to gain insights into the plants’ strategies for improving water use efficiency under different conditions. Furthermore, leaf transcriptomic analysis and soil metagenomics allowed the detection of the interconnectedness between water availability, crop management, and soil health in young vineyards. Overall, our research aims to provide a comprehensive understanding of how grapevines adapt to changing environmental conditions and agricultural practices in order to optimize their photosynthetic performance and overall growth. The results are anticipated to make a valuable contribution to the continuous endeavors aimed at enhancing the resilience of agricultural systems that are capable of flourishing in the context of climate change and environmental sustainability.

## Materials and methods

### Plant material and growth conditions

Grapevine cuttings (*Vitis vinifera* L.) of the tempranillo variety were procured from a certified nursery (Viveros Vitis, Navarra, Spain) and cultivated in a controlled greenhouse setting from April to September 2023. Throughout this period, the greenhouse maintained specific environmental parameters to facilitate optimal growth. The temperature inside the greenhouse was carefully regulated within a range of 25°C–30°C during the daytime and 18°C at night, ensuring a conducive atmosphere for the grapevines. Additionally, a 16-h photoperiod was maintained to mimic natural light conditions, coupled with a relative humidity level of 60%–70% to create a suitable environment for the plants to thrive. The grapevines were planted in 10-L pots filled with well-blended agricultural soil mixed with homogeneous grain quartz sand at a 1:1 volume ratio. Regular and adequate irrigation practices were implemented to support the initial establishment phase of the grapevines for 1 month.

The green cover was a mixture of ryegrass and *T. repens* at a ratio of 1:1 vol:vol. To achieve uniform germination of the seeds, a systematic approach was followed. Initially, a layer of fine quartz sand measuring 0.3 mm was evenly spread on top of the pot’s surface with the cutting previously watered to field capacity. Subsequently, the seeds were distributed uniformly across this sand layer. Then, the seeds were then covered with an additional 0.7-mm layer of sand. Finally, a daily watering regimen was implemented by spraying 150 mL of water onto the surface, ensuring that the seeds received sufficient moisture for their development and growth. The pots without green cover underwent the same process but without seeds. These meticulous steps are crucial in establishing a homogeneous green cover (see **Supplementary Figure S1**).

As soon as the seeds had covered 50% of the crop surface (generally occurring within a period of 3 weeks), the management of the vegetative vigor of the vineyards was carefully regulated to ensure that only one main stem was allowed to flourish. Once the seed cover had reached complete coverage at 100% (generally occurring within a period of 4 weeks), the implementation of treatment addressing soil edaphic water restriction was initiated. Thereafter, sampling was conducted after 2 months.

Leaf samples were harvested 3 h after sunrise in the exact leaf used for gas exchange analysis the day after those analyses. Half of the plant was flash-frozen in liquid N and stored at −80°C until analysis; the other half was used to calculate water content and the mineral analysis.

### Experimental design

The study was conducted using a randomized complete block design with two treatments: well-watered control (field capacity; W) *versus* moderate drought (50% of field capacity; D) and green crop cover (C) *versus* bare soil (NC). Each treatment consisted of six to eight plants for growth parameters and leaf photosynthesis and four times for the other analytics. It is important to note that the leaves neither wilted from the water restriction treatment nor displayed any other noticeable physiological signs.

### Leaf gas exchange analysis

Photosynthetic rates were measured using a portable photosynthesis system (LI-6400XT, LI-COR Biosciences, Lincoln, NE, USA). Measurements were taken 3 h after sunrise under saturating light conditions (1,500 µmol m^−2^ s^−1^) on fully expanded leaves. Parameters measured included maximum net photosynthetic rate (Amax), stomatal conductance (gs), and transpiration rate (E).

### Whole system gas exchange analysis

The gas exchange variation of the entire vineyard–soil system was evaluated using a custom-made gas exchange open system (**Supplementary Figure S2**). The system consisted of a circular chamber of Perspex of 30 L (30-cm diameter, 50-cm height; PLEXIGLAS^®^ XT, Röhm, Germany) made with 29-mm thickness, similar to Jauregui et al. (2018). The Infra-Red Gas Analyse (IRGA) used was a Li-COR Li-7200 (LI-COR); the equipment was set up to gauge every 15 seconds. The chamber was hermetically sealed with Sylgard Silicone Elastomer (Dow Corning, Wales, UK), and each pot was hermetically sealed using a neoprene sponge rubber seal and pressure-sensitive tape. The air circulation was controlled using the Li-COR Li-7200 pump, which allows for a complete chamber turnover in 1 minute. The uniform airflow was confirmed using a smoke test (data not presented). The uniform airflow was confirmed using a smoke test (data not presented). The system was kept at a controlled greenhouse temperature with external air conditioning, ensuring a lower fluctuation of vapor pressure deficit; the plants were kept in this greenhouse for 45 minutes before each measurement.

First, the atmosphere in the enclosure was stirred for 2 minutes using an air current to remove any accumulated moisture. Subsequently, a system was placed inside the enclosure and sealed. The initial 7 minutes was regarded as the stabilization stage; next, the equipment recorded the upcoming 3 minutes. The measurement was derived by deducting the reading of a container with vineyard from a container without vineyard, along with the corresponding verdant covering. Afterward, the overall carbon acquisition and the overall evaporation were calculated by determining the extent of the rise or fall in the measured gas and dividing it by the total plant surface area (vineyard and crop covering), which was evaluated using the smartphone scanner of the application Easy Leaf Area (Easlon and Bloom, 2014).

### ^13^C isotopic composition analyses

Carbon isotope composition (δ^13^C) was measured using an elemental analyzer (EA1108; Carlo Erba Strumentazione, Milan, Italy) linked to an isotope ratio mass spectrometer (Delta C; Finnigan MAT, Bremen, Germany). The δ^13^C values were expressed in ‰ and calculated as δ^13^C = (R_sample_/R_standard_) − 1, where R_standard_ refers to the ^13^C/^12^C ratio of the Vienna Pee Dee Belemnite (V-PDB) international standard.

### Metabolomic analysis

The extraction of primary metabolites was conducted following the protocol outlined by Lisec et al. (2006), using approximately 10 mg of lyophilized leaf tissue previously frozen. Sample derivatization was carried out as described previously (Lisec et al., 2006), and gas chromatography coupled to mass spectrometry (GC–MS) analyses were performed using a 5977C GC/MSD (Agilent, Santa Clara, CA, USA). Metabolite identification was performed manually using the TagFinder software (Luedemann et al., 2012) with reference mass spectra and retention indices obtained from the Golm Metabolome Database (http://gmd.mpimpgolm.mpg.de) (Kopka et al., 2005). The data were normalized by setting the mean value of no cover well-watered (NCW) metabolites to 1, thus providing relative metabolite levels. The data representing the means ± SE from biological replicates correspond to different leaves. The statistical analysis was performed using an ANOVA followed by Tukey’s *post-hoc* tests.

### Hormone analysis

Hormone quantification was carried out using a high-performance liquid chromatography–electrospray ionization–high-resolution mass spectrometry (HPLC–ESI–HRMS) system, which allowed for precise measurement of the hormones in the samples. The extraction, purification, and quantification of indole-3-acetic acid (IAA), abscisic acid (ABA), jasmonic acid (JA), isopentenyladenine (iP), and isopentenyladenine riboside (iPR) were performed following the method outlined by Torres et al. (2018), with slight modifications. Leaf material (15 mg of freeze-dried tissue) was used instead of 0.1 g of frozen powdered tissue. The samples were extracted using an appropriate solvent and then purified by centrifugation and filtration. After evaporation (SpeedVac), the residue was re-dissolved in 0.25 mL of solvent, instead of the original 0.5 mL.

### Leaf transcriptomic analysis

Total RNA was extracted from leaf tissues using the RNeasy Plant Mini Kit (Qiagen, Hilden, Germany; REF MB45601, LOTZQ011) according to the manufacturer’s instructions. RNA quality was assessed using a Bioanalyzer 2100 (Agilent Technologies, Santa Clara, CA, USA). High-quality RNA samples were used to construct cDNA libraries using the TruSeq RNA Sample Preparation Kit (Illumina, USA).

cDNA libraries were sequenced on an Illumina NovaSeq 6000 platform, generating 150-bp paired-end reads. Raw reads were quality-checked using FastQC and trimmed with Trimmomatic. Clean reads were mapped to the *V. vinifera* reference genome using HISAT2, and gene expression levels were quantified using featureCounts. Differential expression analysis was conducted using DESeq2 with EBSeq correction used to increase the resolution (Subramanian et al., 2005; Kharchenko et al., 2014) with the criteria for differentially expressed genes (DEGs): fold change (FC) ≥1.5, false discovery rate (FDR) <0.05. FC is the gene expression ratio. The statistical analysis was performed using BMKCloud (https://www.biocloud.net/).

### Soil microbiome analysis

Soil samples were collected from the root zone of each plant, sieved to remove debris, and stored at −80°C. DNA was extracted using the DNeasy PowerSoil Kit (Qiagen, Germany; REF21802, LOT ZQ031) following the manufacturer’s protocol.

The V3–V4 region of the 16S rRNA gene or ITS1-ITS2 from ITS was amplified using universal primers (341F/806R) and sequenced on an Illumina MiSeq platform, generating 250-bp paired-end reads. Sequence data were processed using the QIIME2 pipeline, including quality filtering, chimera removal, and taxonomic assignment using the SILVA database. The statistical analysis was performed using BMKCloud (https://www.biocloud.net/). Specifically, BMKCloud was utilized to compute alpha diversity indices (such as Shannon, Simpson, and Chao1), and beta diversity metrics based on Bray–Curtis dissimilarity were calculated using QIIME2 (Bolyen et al., 2019), and statistical analyses were performed to identify differences between treatments. Following this, principal coordinate analysis (PCoA), a dimension-reduction technique to visualize differences in species diversity among samples (Gower, 1966), was conducted to illustrate the variations in microbial community composition across samples, allowing for the extraction of key elements and classification of samples to highlight differences in species diversity. To explore how microbial community composition relates to environmental factors like soil pH and moisture, constrained ordination analyses were conducted using redundancy analysis (RDA) for linear responses for unimodal responses based on gradient lengths, with Monte Carlo permutation tests confirming the significance of our findings.

## Results

### Vineyard growth, leaf-level gas exchange parameters, and entire system gas exchange

**Figure 1:**
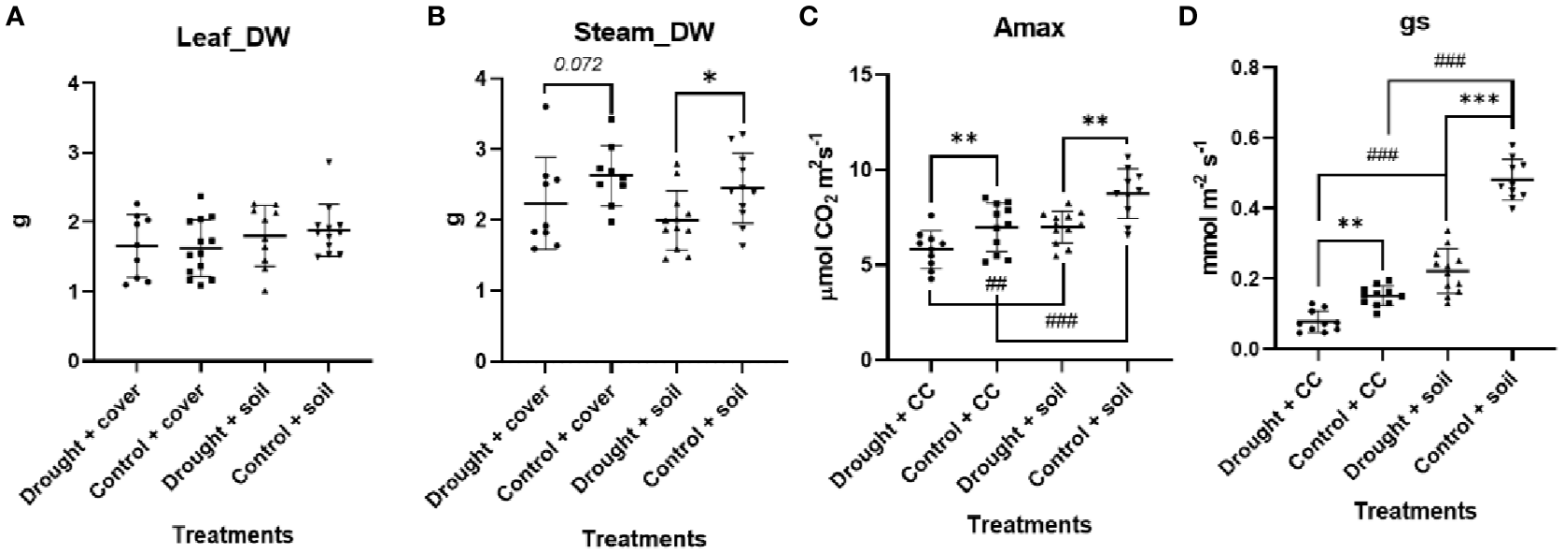
Biomass parameters in vineyard in contrasting crop cover management and edaphic water regime in **(A)** steam dry weight (DW) and **(B)** leaf **(D)**; **(C)** Maximum photosynthesis (Amax) and **(D)** stomatal conductance (gs). Individual observations, the average, the standard deviation, and q-values are represented. Black bars, dots, and error bars represent statistical power: Welch’s ANOVA test and false discovery rate of one step-up procedure of Benjamini, Krieger, and Yekutieli (*n* = 9–12). Asterisks denote levels of statistical significance: * for *p* < 0.05, ** for *p* < 0.01, and *** for *p* < 0.001. Number sign denote levels of statistical significance: # for *p* < 0.05, ## for *p* < 0.01, and ### for *p* < 0.001.

The findings suggest that the use of crop cover does not have a statistically significant impact on the accumulation of biomass in both stems and leaves (**Figure 1**). Thus, the imposition of water stress leads to a notable decrease in the allocation of biomass to stems when grown in bare soil, while it is not significant for crop cover (*p* = 0.07). It is noteworthy that the trends observed in stem biomass allocation are not mirrored in leaf biomass allocation, as plants have similar leaf biomass across all experimental conditions.

**Figure 2:**
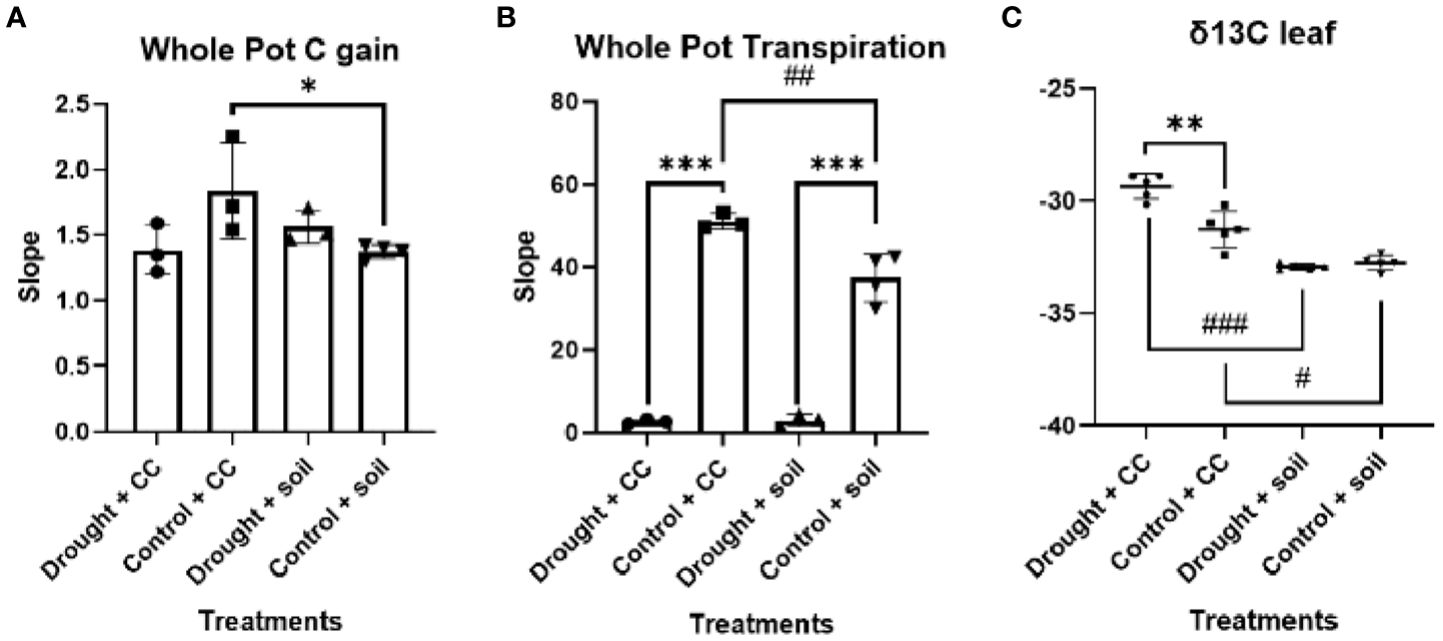
Whole pot gas exchange and δ^13^C. Data represent the slope of **(A)** carbon gain and **(B)** transpiration for 120 seconds selected into the linear phase. In panel **(C)**, data represent δ^13^C. Individual observations, the average, the standard deviation, and q-values are represented. Black bars, dots, and error bars represent statistical power: Welch’s ANOVA test and false discovery rate of one step-up procedure of Benjamini, Krieger, and Yekutieli (**A** and **B**, *n* = 3; **C**, *n* = 5). Asterisks and number sign denote levels of statistical significance: * or # for *p* < 0.05, ** or ## for *p* < 0.01, and *** or ### for *p* < 0.001.

As expected, the water restriction treatment leads the gs in these leaves to be limited (**Figure 1**). Furthermore, there was a reduction in the fully expanded leaf Amax compared to the control conditions under both types of soil cover treatments. Additionally, it was also observed that leaf Amax exhibited notably higher values in vineyards grown in bare soil compared to those utilizing cover crops. The difference in Amax can be attributed to the significantly higher gs observed with this soil treatment. When evaluating the complete soil-plan consortium (**Figure 2**), pots with cover crops demonstrated greater total carbon accumulation in well-watered environments, attributed to their widespread coverage and enhanced photosynthetic surface area. However, evapotranspiration notably rose under well-watered conditions, reaching its highest point for WC.

### Vineyard leaf ^13^C isotopic composition

The response of vineyard leaves δ^13^C to limited water conditions was observed to be both robust and consistent in our study (**Figure 2**). An intriguing finding of our study was that vineyards in bare soil exhibited greater discrimination against δ^13^C, yet no distinctions were observed among them.

### Vineyard leaf transcriptomic analysis

**Figure 3:**
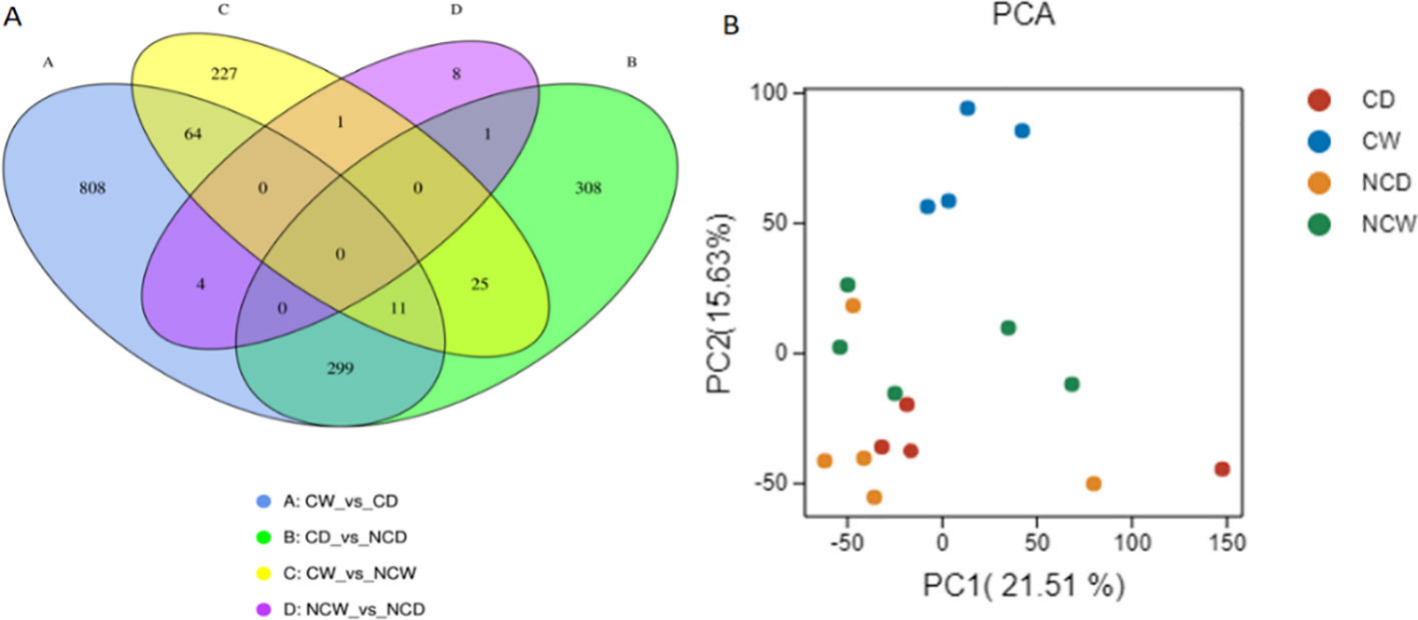
Represents clustering of transcripts along the treatments into **(A)** a Venn Diagram, and **(B)** a PCA plot (*n*=4).

The findings indicate that the leaf transcriptome experiences considerable changes as a result of the treatments. The Venn diagram (**Figure 3A**) illustrates the variations in gene expression or presence across different comparisons. Remarkably, NCW_*vs*_NCD displayed a strikingly comparable transcriptome pattern, underlying its similarities. Principal component analysis (PCA) was conducted on the fragments per kilobase of transcript per million mapped reads (FPKM) of each sample in order to uncover patterns and relationships within the data (**Figure 3**). The PCA analysis (PC-1, 21.51%; PC-2, 15.63%) revealed distinct clustering of plants: CW clustered together, while other treatments showed increased dispersion. CW clustered separately from CD, while this pattern was less clear for treatments with no cover. Such differences are related and illustrated in the volcano plots (**Supplementary Figures S3A–E**).

Enrichment analysis based on EuKaryotic Orthologous Groups (KOG) offers phylogenetic classification and orthologous information for proteins, helping to unveil key biological pathways, molecular mechanisms, and core regulatory elements affected by the treatments (**Supplementary Figure S2**). In the W_*vs*_D comparison, the analysis revealed limited roles (>10% frequency) of functional classes including defense mechanisms, inorganic ion transport and metabolism, secondary metabolite biosynthesis, general functions, amino acid transport, and signal transduction. For the C_*vs*_NC comparison, carbohydrate transport and metabolism showed predominant roles (>30% frequency), followed by signal transduction mechanisms, general functions, and posttranscriptional regulations (>20% frequency). The CW_*vs*_CD comparison revealed profound impacts (>50% frequency) in carbohydrate transport and metabolism, posttranscriptional modifications, cell cycle control, general functions, defense, cell wall/membrane/envelope biogenesis, and signal transduction. The CD_*vs*_NCD comparison showed profound impacts on carbohydrate transport and metabolism, signal transduction, and general functions, with relevant roles in multiple groups including inorganic ion transport, posttranscriptional modifications, secondary metabolism, amino acid transport, and lipid metabolism.

By classification of the number of DEGs into the Gene Ontology (GO), the results emphasize the synergistic advantages of cover crops and water availability, along with the limitations caused by the absence of cover crops (**Supplementary Figure S3**). In the W_*vs*_D comparison, biological processes showed moderate enrichment in D, primarily in pathways related to cellular responses, metabolic activities, and stress responses. Cellular component analysis indicated the significant engagement of membrane-associated genes in D, suggesting active membrane remodeling or transport processes. Conversely, the C_*vs*_NC comparison showed a more pronounced transcriptional response, with biological processes indicating a greater number of engaged genes, particularly within primary metabolism and cellular activities.

Then, we examined the categorization of DEGs within the Kyoto Encyclopedia of Genes and Genomes (KEGG) pathways, which offered a deeper understanding of the transcriptomic response (**Supplementary Figure S4**). In the W_*vs*_D analysis, pathways associated with photosynthesis and carbon metabolism were significantly downregulated under D, while pathways related to plant hormone signaling, fatty acid elongation, and glycerolipid and glycerophospholipid metabolism were upregulated under D, reflecting enhanced signaling and membrane-related activities that likely facilitate adaptation to drought. In the C_*vs*_NC comparison, phenylpropanoid and flavonoid biosynthesis pathways were upregulated in C, indicating the heightened synthesis of secondary metabolites that may contribute to defense mechanisms, while fatty acid elongation and tyrosine metabolism were downregulated. Plant–pathogen interactions and phytohormonal signaling demonstrated mixed regulatory responses, highlighting the complex physiological adjustments to variations in plant cover. In the CW_*vs*_CD analysis, the CW condition showed significantly higher enrichment in essential biological pathways, particularly flavone and flavanol biosynthesis, ABC transporters, and phenylpropanoid biosynthesis, suggesting greater resource allocation for defense-related secondary metabolites. CW treatment also showed improved vitamin B6 metabolism. The CW_*vs*_NCW comparison highlighted the beneficial role of cover crops under well-watered conditions, with NCW showing increased enrichment in glyoxylate and dicarboxylate metabolism, carotenoid biosynthesis, glycine–serine–threonine metabolism, flavonoid biosynthesis, arginine metabolism, zeatin biosynthesis, and nitrogen and carbon metabolism, indicating specific adaptations through strategic resource redistribution. In the CD_*vs*_NCD comparison, vineyards grown with crop cover showed enrichment in phenylalanine metabolism, linoleic acid metabolism, beta-alanine, isoquinoline alkaloid biosynthesis, and phagosome, while brassinosteroid biosynthesis and thiamine were enhanced in bare soil.

### Primary metabolite and hormone profiling

**Figure 4:**
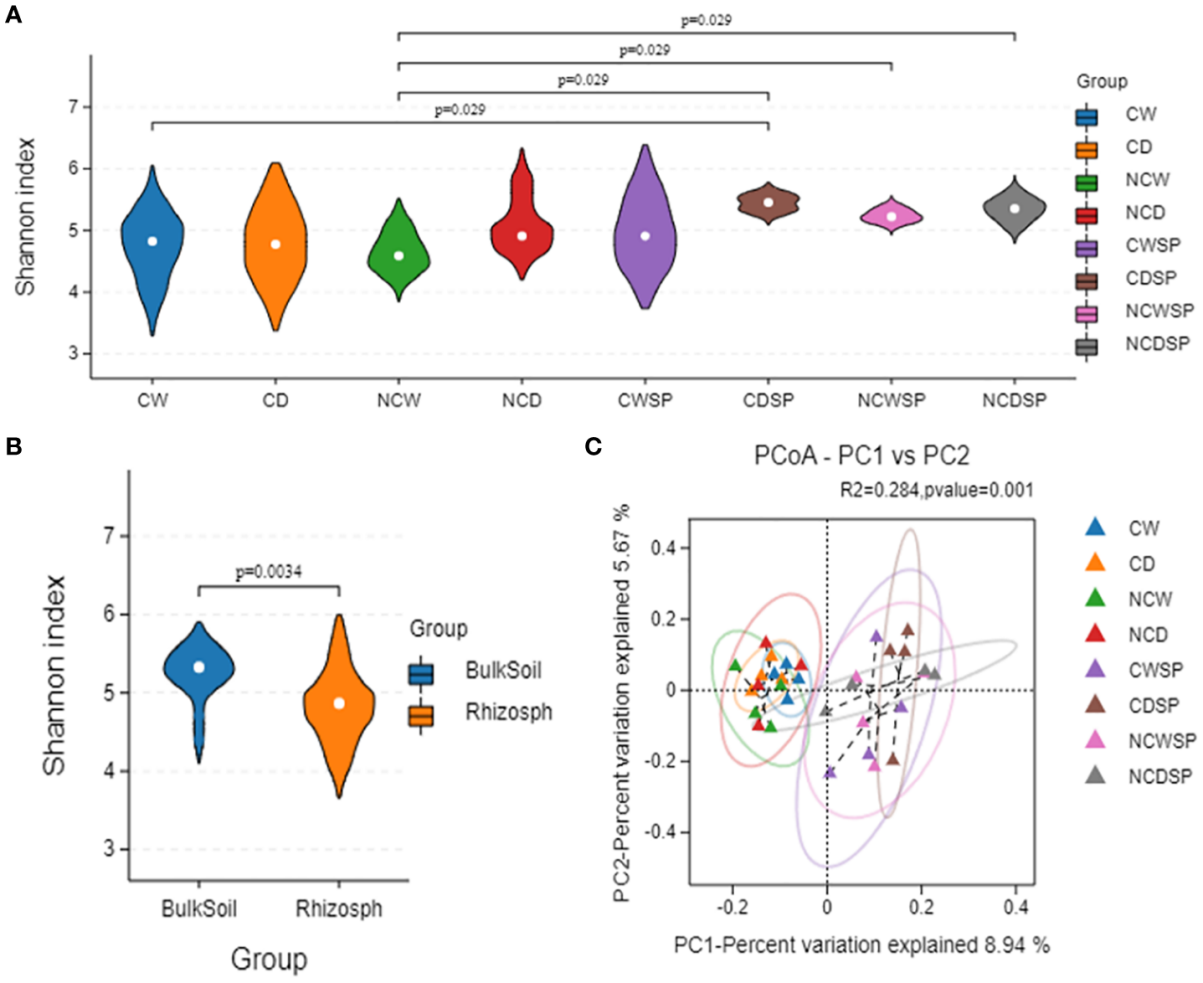
Represents bacterial alpha and beta diversity of soil microbiota represented in **(A, B)** Shannon index, and **(C)** PCoA plot (*n*=4).

The primary metabolite profiling revealed significant variations across the four different treatments (**Figure 4**). The findings clearly demonstrate that the presence of cover crops plays a pivotal role in promoting the accumulation of various leaf sugars including fructose, glucose, xylose, galactinol, trehalose, fucose, sucrose, raffinose, rhamnose, and isomaltose. Additionally, under drought conditions, the accumulation of several sugars and polyols (i.e., fructose, galactinol, myo-inositol, isomaltose, raffinose, and xylose) was notably higher compared to irrigated plants in covered plants. However, drought only induced moderated increases in some sugars (fructose, glycerol, and raffinose) in non-covered plants.

Regarding organic acids, only a significant accumulation in citrate, among all tricarboxylic acid (TCA) cycle intermediates detected, was observed in covered plants. As for sugars, citrate was further increased under drought conditions, with levels becoming more than twice those observed in bare soil (**Figure 4**). Beyond this step, the levels of most TCA cycle intermediates were not changed by the cover presence, except for succinate, which exhibited increased levels in vineyards with crop cover under irrigated conditions. In addition, 2-oxoglutarate displayed lower levels in covered plants. The organic acids related to ascorbate metabolism showed significant changes, with both ascorbate and dehydroascorbate displaying increased levels under both crop cover scenarios (CW and CD). Conversely, threonate levels (a product from ascorbate-related metabolites) were decreased in vineyards cultivated with crop cover compared to those in bare soil. Notably, the levels of shikimate were lower in covered plants, particularly under drought, while glycerate showed higher levels under drought.

Regarding amino acids, glycine and tryptophan exhibited significantly greater accumulation in both crop cover treatments (CW and CD), as compared to non-covered plants (NCW and NCD), particularly under drought conditions (**Figure 4**). Also, aspartate and glutamate exhibited markedly increased levels under drought in covered plants (CD) compared to the other conditions, thus indicating that these amino acids may play a specific role in the response to drought stress when crop cover is present. Lastly, other metabolites such as GABA and phosphoric acid showed decreased levels across all treatments, whereas urea displayed highly variable results (**Figure 4**).

Concerning the hormone levels (**Table 1**), IAA exhibited a significantly lower concentration in NCD compared to CD. ABA demonstrated the most substantial variations among the treatments. Initially, as anticipated, it is notably elevated in CD relative to CW and reaches its peak in CD compared to NCD. Moreover, unexpectedly, the hormone associated with drought stress displayed no significant differences in bare soil across the water treatments and was found to be higher in CW than in its counterpart in bare soil. JA revealed a significant difference in the CW_*vs*_NCW comparison, being more concentrated in bare soil. The only cytokinins that indicated variations were iP and iPR. To begin with, iP levels are significantly elevated under drought stress, but only when crop cover is present in the system; additionally, as it showed the highest concentration, CD content surpasses that of NCD. Finally, iPR exhibited notable differences in the CW_NCW comparison, being more abundant with crop cover in the system.

**Table 1:** The phytohormone content in pmol/gPS and statistical comparison across treatment conditions including auxin (IAA), abscisic acid (ABA), jasmonic acid (JA), isopentenyladenine (iP), and isopentenyladenosine (iPR).

(pmol/gPS)	IAA	ABA	JA	iP	iPR
CW	1,563 ± 5.5	3,297.7 ± 294.2	35.2 ± 5.2	4.1 ± 0.1	1.3 ± 0.1
CD	155.3 ± 2.9	5,802.0 ± 179.4	87.6 ± 34.8	5.4 ± 0.3	1.6 ± 0.2
NCW	143.9 ± 4.0	2,132.3 ± 60.1	67.3 ± 10.7	3.6 ± 0.2	0.9 ± 0.0
NCD	135.1 ± 7.9	2,626.1 ± 278.9	89.3 ± 16.6	3.7 ± 0.3	1.5 ± 0.4
Variable	IAA	ABA	JA	iP	iPR
CW_*vs*_CD	ns	***	ns	***	ns
NCW_*vs*_NCD	ns	ns	ns	ns	ns
CW_NCW	ns	**	*	ns	**
CD_*vs*_NCD	*	***	ns	**	ns

The treatment comparison includes cover well-watered (CW), cover drought (CD), no cover well-watered (NCW), and no cover drought (NCD), with a replication of n = 4.

* *p* < 0.05; ** *p* < 0.01; *** *p* < 0.001; ns, no significance.

### Microbial community composition and diversity

#### Operational taxonomic unit analysis

Operational taxonomic unit (OTU) analysis (**Supplementary Figures S5A, B**) demonstrated that bacterial communities largely overlapped across treatments. Soil plant cover had a marginal impact, while soil water content showed 2,057 common OTUs, with 141 unique to well-watered conditions and 77 to drought. In contrast, vineyard presence resulted in more pronounced differences, with only 495 shared OTUs, 251 unique to vineyard rhizosphere, and 156 unique to non-vineyard soil. The presence of vineyard plants markedly enhances bacterial community diversity. Unweighted Pair Group Method with Arithmetic Mean (UPGMA) clustering using the binary Jaccard method revealed that bulk soil samples cluster distinctly from rhizospheric soil, underscoring the vineyard’s influence (**Supplementary Figures S5C, D**).

#### Alpha and beta diversity

**Figure 5:**
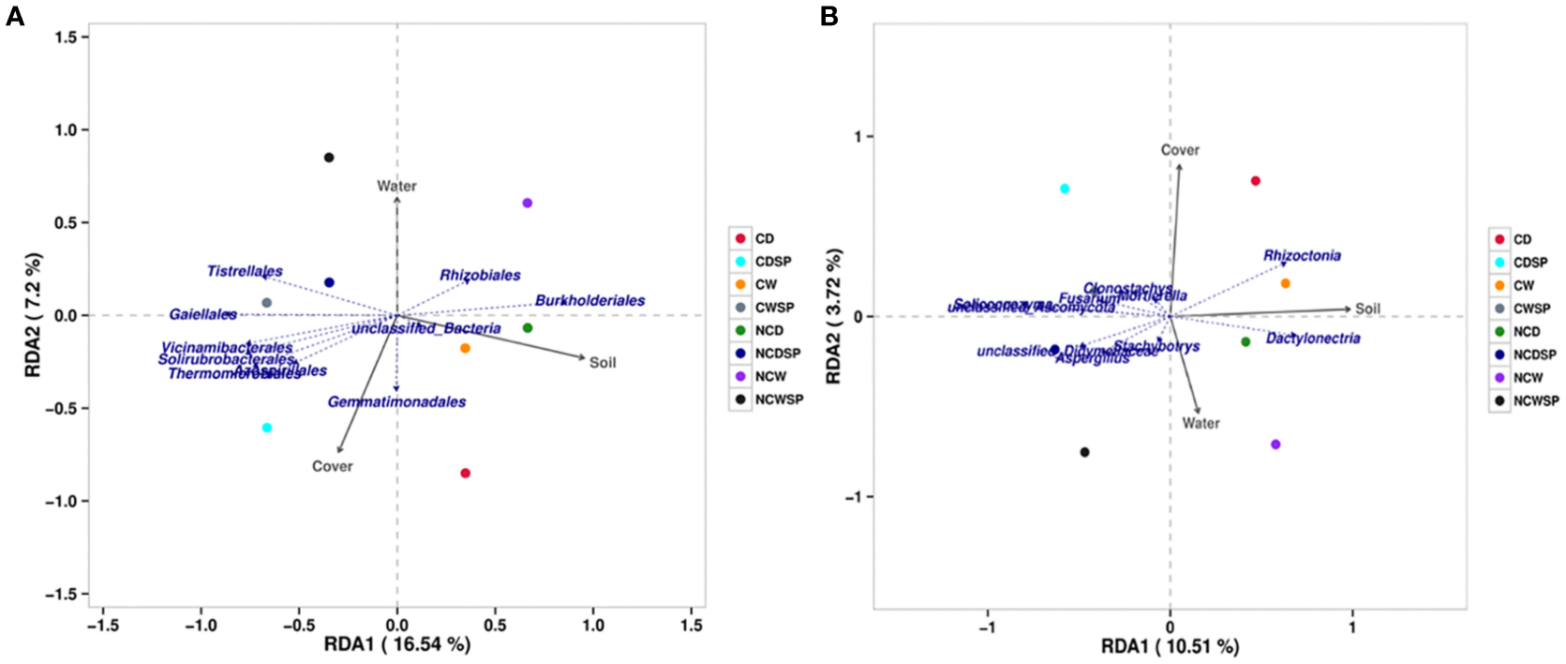
Represents RDA ordination in **(A)** microbial and **(B)** fungal communities (*n*=4).

Alpha diversity metrics (Shannon, Simpson, and Chao1; **Figure 5**) showed significant variations in microbial diversity under different treatments. First, as expected, utilizing the Abundance-based Coverage Estimator (ACE; *p*-value 0.003), Chao-1 index (*p*-value 0.009), Shannon (*p*-value 0.0021), and Simpson (*p*-value 0.0083) indices, results indicated that vineyards influence bacterial microbiota, with higher diversity without vineyard in the system. Microbial community diversity was significantly lower in bare soil and well-watered conditions than in others (Shannon *p* = 0.023; Simpson *p* = 0.037). Bulk soil groups consistently demonstrated the greatest diversity, whereas NCW exhibited the lowest diversity values.

Beta diversity, assessed via PCA and PCoA with the Bray–Curtis dissimilarity, demonstrated distinct separation of microbial communities based on vineyard presence and crop cover. Specifically, rhizospheric soil from vineyards clustered separately from non-vineyard soils (**Figure 5B**), and the combination of crop cover with well-watered conditions (PC-1, 23.64%; PC-2, 8.33%; **Figure 5C**) yielded more uniform communities. Notably, drought induced more unique microbial profiles in bare rhizospheric soil, whereas well-watered conditions resulted in similar communities regardless of cover.

#### Taxonomic composition and differential abundance

At the phylum level (**Supplementary Figure S6A**), predominant groups such as Chloroflexi, Geminicoccaceae, *Rubrobacter*, and *Paucibacter* were identified. *Paucibacter* was notably more abundant in vineyard rhizospheres and under crop cover. Differential analysis via ANOVA revealed that Proteobacteria were enriched in bare vineyard soil, whereas Actinobacteria, Acidobacteria, Chloroflexi, and Gemmatimonadota were reduced in bare vineyard rhizospheres compared to covered soils; conversely, Patescibacteria increased under these conditions. At the order level (**Supplementary Figure S6B**), Burkholderiales were significantly more abundant in vineyard rhizospheres under bare soil compared to crop-covered conditions, while Vicinamibacterales were more prevalent in bulk soil. Gemmatimonadales were reduced in CD treatments. Other orders (e.g., Solirubrobacterales, Tistrellales, Gaiellales, Thermomicrobiales, Microtrichales, Rubrobacterales, Actinobacteriota, Micrococcales, and Acetobacterales) showed decreased abundance in bare vineyard rhizospheres relative to covered ones but remained stable in non-vineyard soils.

**Figure 6:**
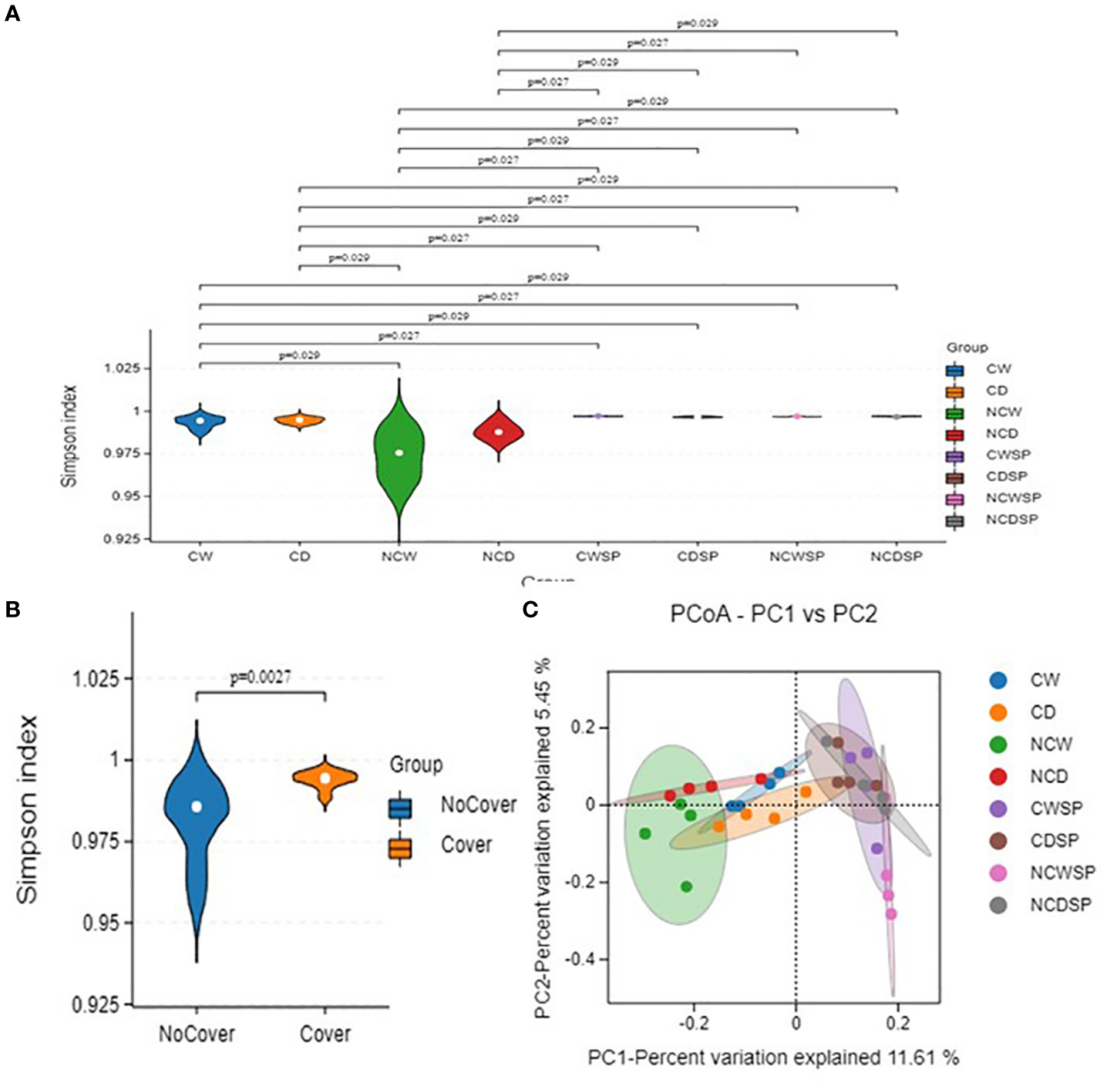
Represents fungal alpha and beta diversity in soil fungal communities in **(A, B)** Shannon index, and **(C)** PCoA plot (*n*=4).

RDA (**Figure 6A**) revealed that environmental factors explained 23.74% of the bacterial community variation. Burkholderiales and Rhizobiales showed positive associations with vineyard presence and water content, while Tistrellales (and Gaiellales) were negatively correlated with vineyards and positively associated with crop cover. A cluster of Vicinamibacterales, Solirubrobacterales, and Thermomicrobiales was strongly linked to crop cover and inversely related to vineyard presence and water content. Gemmatimonadales exhibited a moderate affinity for crop cover with a slight drought correlation.

### Fungal community composition and diversity

#### Operational taxonomic unit analysis

**Figure 7:**
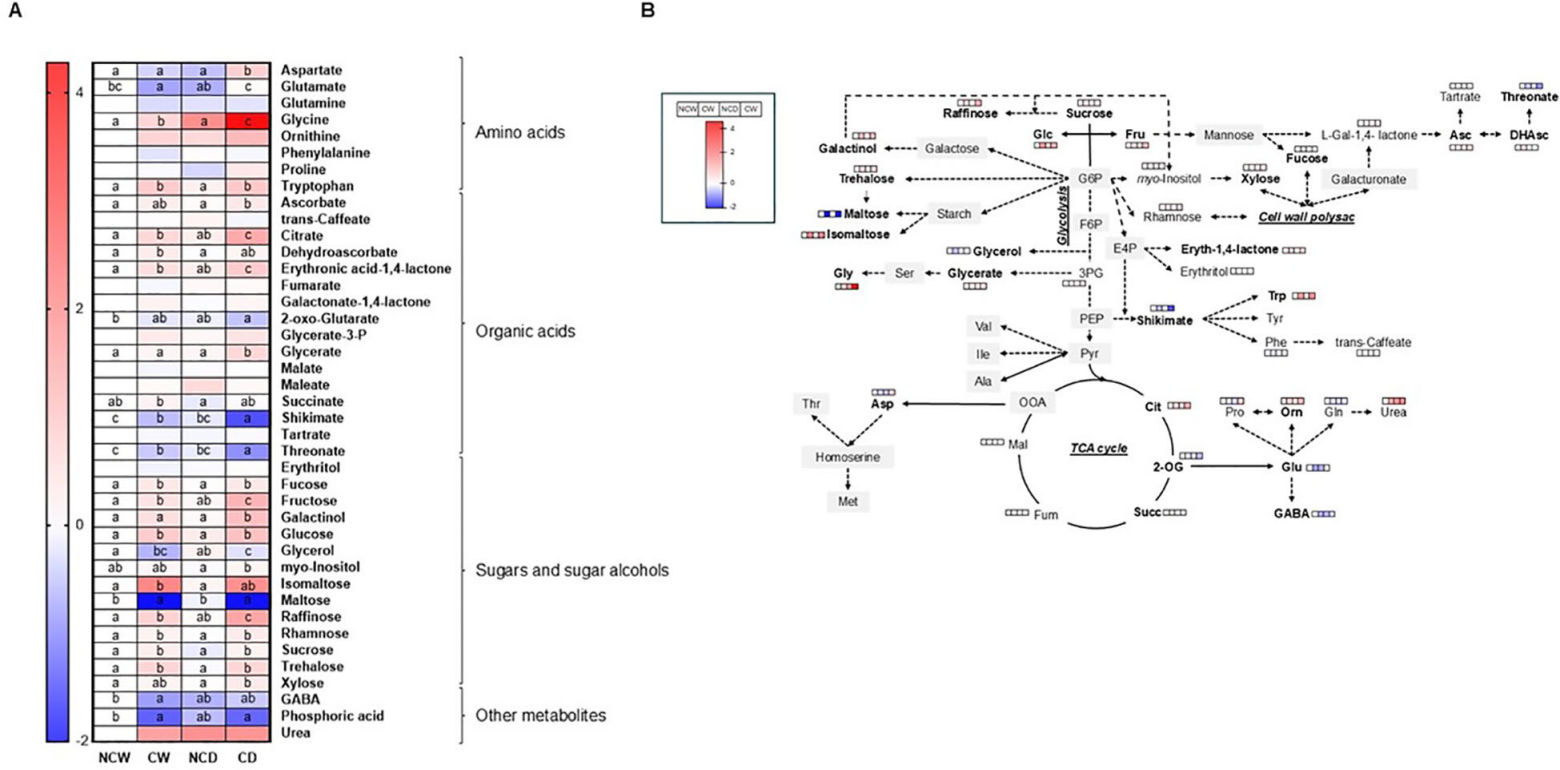
Represents **(A)** heatmap of the leaf metabolomics and **(B)** pathway diagram representation (*n*=4).

Fungal OTU analysis (**Figure 7**) (**Supplementary Figure S7**) highlighted more pronounced differences compared to bacteria. Crop cover influenced fungal communities, with 364 shared OTUs, 156 unique to crop cover, and 165 unique to bare soil. Soil water content yielded 379 common OTUs, with 157 unique to optimal watering and 149 unique to drought. Vineyard presence resulted in 490 shared OTUs, 159 unique to the vineyard rhizosphere, and 182 unique to non-vineyard soil, as further illustrated by the flower diagram (unique OTUs ranging from 24 to 38).

#### Alpha and beta diversity

Fungal alpha diversity (**Figure 7**) (**Supplementary Figure S7A**) showed that, in the absence of a vineyard, bulk soil exhibits higher diversity—particularly in the CD, NCW, and NCD treatments—with a higher Shannon index compared to rhizospheric soil. Beta diversity analysis using PCoA with binary Jaccard indicated clear separation between vineyard-associated and non-vineyard fungal communities, with no other distinct clustering observed.

#### Taxonomic composition and differential abundance

Hierarchical clustering of fungal communities (**Supplementary Figure S8**) revealed substantial compositional variation. Predominant phyla included *Fusarium*, *Rhizoctonia*, *Stachybotrys*, and *Mortierella*, along with significant proportions of unclassified fungi (notably from the Didymellaceae family). Additionally, *Aspergillus* was more abundant in non-vineyard soils, whereas *Dactylonectria* was higher in vineyard rhizospheres.

### Differential abundance

ANOVA-based differential abundance analysis showed that plant cover significantly affects fungal community composition. At the order level, dominant taxa included Cantharellales, Pleosporales, Pezizales, Filobasidiales, and unclassified Ascomycota. Cantharellales were enriched in covered treatments, Pezizales were lower in CW compared to other vineyard groups, and unclassified Ascomycota exhibited the opposite trend. At the family level, *Rhizophila* was the most abundant (~0.25), particularly in CW and NCW, whereas lower-abundance families such as *Dactylonectria* increased in rhizospheres, and *Penicillium* and *Pseudeurotium* were more prevalent in non-vineyard soils.

Fungal RDA (**Figure 6B**) explained 14.23% of the community variation (RDA1, 10.51%; RDA2, 3.72%) and revealed that *Rhizoctonia* is strongly associated with vineyard presence (notably in CD and CW), while *Dactylonectria* correlates positively with vineyards but negatively with well-water conditions. *Clonostachys* showed a moderate association with crop cover, whereas *Helicodendron* and *Xenochalara* were negatively linked to soil properties yet moderately connected to crop cover. *Aspergillus*, located in the negative quadrant of RDA1, exhibited weak-to-moderate associations with water content and soil. Overall, fungal responses were less pronounced than bacterial ones, yet they exhibited distinct treatment-specific environmental associations.

## Discussion

Cover cropping in viticulture has recently emerged as a sustainable management approach: it is evolving from the traditional soil conservation method (Unger and Vigil, 1998) to a multifunctional tool that tackles various challenges including ecosystem services, increased soil microbiome diversity, and resilience against climate change (Steenwerth and Belina, 2008; Lamichhane and Alletto, 2022). According to previous studies, yield variations related to cover cropping show significant variability, with research showing reductions under water-limited conditions (Ruiz-Colmenero et al., 2011; Wang et al., 2021), whereas neutral beneficial effects are noted in regions with adequate water conditions (Abad et al., 2021b). Furthermore, as cover crops can reduce vineyard vegetative growth under water-imitating conditions (Fleishman et al., 2023), special care must be taken in implementing crop covers in rain-fed Mediterranean vineyards. At the moment, crop cover management is seen as a sustainable approach to address drought stress only in the long term, thanks to its benefits in improving soil water retention and infiltration by enhancing organic matter and structure (Zhang et al., 2023). In this scenario, our experiment represents a significant milestone because crop cover mitigated drought stress effects faster than anticipated in the bibliography, as seen in our 3-month experiment (i.e., a lack of significant reduction in stem biomass was observed in covered plants; *p*-value <0.072; **Figure 2**). The combination of various snapshots and time-integrating physiological methodologies enabled us to explain this striking result. At the leaf level, our photosynthetic analysis indicates that crop cover significantly limits vineyard carbon fixation capacity, primarily due to stomatal constraints. The slight decrease in stomatal conductance under Crop Cover (CC) likely reflects a precise balance between water conservation and CO_2_ uptake. Nevertheless, it is noteworthy that it did not lead to a statistically significant alteration in the overall vineyard carbon gain under drought (**Figure 2**). While the use of cover crops resulted in a notable increase in system-wide evapotranspiration (**Figure 2**), the δ^13^C results reveal that it is indeed the vineyard in bare soil that denotes carbon isotopic fingerprint modification, with greater discrimination against the heavier carbon isotope during photosynthesis (Farquhar and Richards, 1984), indicating vineyards in bare soil accumulate stress. Therefore, this finding indicates that contrary to our hypothesis, crop cover indeed exerts a beneficial effect on vineyards under drought. Our research indicates that the use of cover crops can provide quick advantages in alleviating drought stress—benefits that were previously thought to develop gradually over time. Throughout our 3-month study, the lack of a noticeable decrease in stem biomass among vines with cover crops suggests that these plants can help sustain vine health even in water-limited situations. Furthermore, the modest decline in gs observed in CC under drought likely represents an optimal trade-off between conserving water and facilitating CO_2_ uptake. These swift reactions imply that, in areas experiencing more frequent and severe droughts due to climate change, cover crops could act as an effective, short-term adaptive measure to enhance water retention and soil quality. For effective vineyard management, our results strongly encourage further testing under actual field conditions to validate our promising findings: particularly in rain-fed Mediterranean environments, CC can bolster soil moisture retention and infiltration, thus enhancing the vineyard’s ability to withstand water-limiting conditions in the early stage.

The integration of multi-omics with physiological characterization offers insight into how crops adapt to stress (Aranjuelo et al., 2015; Zargar et al., 2022; Florez-Sarasa et al., 2020). In our experiment, this has helped us to capture particular relationships in the dynamic interplay between the availability of water resources and the crop cover treatment in vineyards’ metabolism. Our results clearly indicate that drought influences resource distribution. Under drought, we found that the transcription of photosynthesis and carbon metabolism pathways is notably downregulated (**Figure 3**), which correlates with the observed declines in leaf-level photosynthetic rates. This finding correlates with the transcriptomic analysis, indicating large changes in gene expression related to carbohydrate transport and metabolism (>60% frequency in the CW_*vs*_CD comparison). Furthermore, our data indicated a notable build-up of sugars and sugar alcohols in both covered and non-covered plants under drought, such as fructose, glycerol, and raffinose, which function as osmolytes, preserving cellular integrity and ensuring osmotic equilibrium to cope with water stress (Zandalinas et al., 2022). Remarkably, additional increases in raffinose family oligosaccharides (galactinol and myo-inositol), isomaltose, and xylose were particularly observed in vineyards under crop cover, and basal levels of several sugars and sugar alcohols were higher in covered plants (fructose, glucose, xylose, galactinol, trehalose, fucose, sucrose, raffinose, rhamnose, and isomaltose). Altogether, these results suggest that cover treatment induced some priming and enhanced metabolic effect towards drought stress. In line with this, increased levels of glycine and tryptophan under both watering conditions, as well as those of aspartate under drought, could also be beneficial for the drought response of covered plants. These results were also aligned with the transcriptomic response showing enhanced pathways for amino acid transport and metabolism (especially in the CW_*vs*_CD comparison). Changes in tryptophan metabolism have been linked to ABA biosynthesis (Yoshida and Fernie, 2024), in addition to being an auxin precursor. In agreement with these observations, the levels of IAA and ABA were higher in covered *vs*. non-covered plants under drought (**Figure 4**). Plant hormone signaling and fatty acid elongation pathways, which may prove beneficial in aiding the plant’s resilience in the face of water limitations with crop cover, were upregulated (**Figure 4**; **Table 2**) with crop cover. The metabolomic data from leaves corroborate these transcriptional changes. The hormone analyses corroborate these transcriptional changes. Crop cover seems to play an essential role in regulating vineyard reactions to drought by promoting beneficial hormonal interactions. ABA levels were unexpectedly lower in bare soil, emphasizing crop cover’s role in enhancing vineyards’ drought resilience and response, which could be related to metabolic priming effects (Schwachtje et al., 2019). Cytokinins like iP and iPR showed greater concentrations under crop cover, indicating a potential influence on hormonal balance, while bare soil exhibited higher JA levels, emphasizing that crop cover indeed provides a crucial protective mechanism for vineyards under water scarcity, fostering resilience within a short period; previous studies have shown that cover crops and their residues facilitate root penetration through compacted soil, allowing cash crops to access deeper water reserves in the long term (Yang et al., 2021). Furthermore, the amino acid metabolism also indicates its potential role in mitigating drought stress: the large content of aspartic acid and its derivate in CD, together with the elevated content of glycine and tryptophan found in both crop cover treatments (especially in the CW_*vs*_CD comparison), aligns with the transcriptomic response showing enhanced pathways for amino acid transport and metabolism. Such amino acids are well-known metabolites (Zandalinas et al., 2022; Li et al., 2024). Finally, it is noteworthy that the vineyards with crop cover showed an upregulation of pathways involved in the biosynthesis of secondary metabolites, such as phenylpropanoids and flavonoids, which may be contributing to an enhanced defense and protection response. In line with this, the levels of shikimate, the critical intermediate connecting primary metabolism with the mentioned secondary metabolism pathways, were markedly lower in covered plants, particularly under drought. In clear contrast, bare soil appeared to hinder the plant’s ability to effectively utilize these adaptive responses—with no major differences in NDW_*vs*_NCD—resulting in notable impacts on leaf physiology and overall productivity mentioned above. Summarizing, our research demonstrates previously unobserved plant–plant interactions in which crop cover initiates a systematic adaptation process in vineyards, which boosts vineyards resilience to soil water stress by promoting metabolite accumulation and alterations in gene expression.

**Table 2:** Differentially expressed gene (DEG) analysis on vineyard leaves across multiple treatment comparisons across three major annotation databases: EuKaryotic Orthologous Groups (KOG), Gene Ontology (GO), and Kyoto Encyclopedia of Genes and Genomes (KEGG) pathways.

DEG set	Total	KOG	GO	KEGG
Control_*vs*_Drought	167	93	143	128
Cover_*vs*_NoCover	411	212	344	291
CW_*vs*_CD	1,168	644	990	860
CD_*vs*_NCD	633	311	549	479
CW_*vs*_NCW	316	158	261	213
NCW_*vs*_NCD	14	8	13	11

The comparisons include control vs. drought, cover vs. no cover, and various combinations of treatments labeled as cover well-watered (CW), cover drought (CD), no cover well-watered (NCW), and no cover drought (NCD), with a replication of n = 4.

It has been proposed that vegetation cover provides stable microhabitats and resources that sustain key functional microbial communities (Li et al., 2024). The analysis of the microbial community further supports this observation: the RDA ordination analysis underscores the major impact of crop cover on community structure (RDA1 8.94% *vs*. RDA2 5.67% for bacteria; RDA1 11.61% *vs*. RDA2 5.45% for fungi). Furthermore, we found that the impact of crop cover on microbial population even surpasses the impact of water limitation; Domeignoz-Horta et al. (2024) found that vegetation has a greater impact on soil microbiome resilience than water availability in agricultural ecosystems. However, detecting specific taxa is essential, as ecosystem resilience to drought can be influenced by particular microbial taxa (Metze et al., 2023). In our case, some identified potential candidates may explain the overall vineyard fitness. First, Tistrellales and Gaiellales, the orders that contribute to organic N recycling (Renaud-Martins et al., 2023), exhibit substantial correlations with crop cover under control, indicating potential benefits for the health of vineyards. Conversely, Gemmatimonadales, although not classified as plant-associated microorganisms, contribute to soil nutrient recycling ecosystems (Du et al., 2024) and may therefore also be vital for the responsiveness of vineyards in these conditions. Regarding the fungal populations, the presence of vegetative cover significantly influences the properties of rhizospheric soil to a greater extent than bacterial populations, results that are in line with many others (Lehman et al., 2012; Muturi et al., 2024). Thus, the abundance of the fungal community under crop cover may create a more stable ecosystem, which could foster more reliable microbial interactions and enhance drought resistance. *Rhizoctonia*, known to affect plant development and disease risk through its interactions with other microbes (Whipps, 2001), showed a strong correlation with rhizospheric soil of vineyards experiencing drought when crop cover is present. In conjunction, *Dactylonectria* supports vineyard soils during drought conditions but is found in lower quantities in well-irrigated environments, indicating a specialization for drought resilience, which could be linked to overall plant vitality (Longone et al., 1996). Taking a closer look, our results imply a complex interaction between vineyards and microbes, particularly stress response pathways, potentially enhancing nutrient uptake.

## Conclusions

Our research indicates that implementing cover crops provides surprising short-term advantages for enhancing drought resilience for vineyards. The study indicated that despite leaf-level photosynthetic measurements, there was an increase in overall evapotranspiration during drought conditions when cover crops were utilized, yet it unexpectedly sustained carbon accumulation in vineyards. Metabolomics revealed elevated levels of osmolytes and stress-related metabolites (including sugars, sugar alcohols, and amino acids) in vineyards with cover crops, along with elevated ABA concentrations in vineyards of crop cover under drought when compared to other conditions. Transcriptomic demonstrates a comprehensive molecular adjustment to drought in vineyards with crop cover. Drought conditions led to the downregulation of photosynthetic and carbon metabolic pathways, matching with metabolomics. Furthermore, cover cropping enhanced the expression of defense-related pathways, particularly those involving plant hormone signaling and secondary metabolites. Conversely, vineyards without cover crops exhibited limited transcriptional responses, indicating that cover cropping facilitates a more robust drought resilience. An analysis of the microbial community showed that the presence of crop cover had a more significant effect on community composition than drought. Certain taxa exhibited responses that depended on the treatment, with Tistrellales and Gaiellales being associated with crop cover under optimal conditions, while *Rhizoctonia* displayed a strong link to rhizospheric soil under drought conditions when crop cover was present. To the best of our knowledge, this is the first time that results collectively illustrate that cover cropping can improve cash crop resilience against drought stress through intricate interactions among plants, soil, and microbes, providing benefits from early implantation.

## Data Availability

The amplicon sequencing data presented in the study are deposited in the Figshare repository, accession number: doi: 10.6084/m9.figshare.29086661.

## Supplementary material

Supplementary Figure 1 Image of the implementation of the crop cover into the experiment; picture taken 1-week previous to apply the water treatment.

Supplementary Figure 2 Enrichment analysis based on cluster KOG.

Supplementary Figure 3 Enrichment analysis based on cluster GO.

Supplementary Figure 4 Enrichment analysis based on cluster KOG.

Supplementary Figure 5 Clustering of the samples **(A)** using flower or Venn diagrams; and using UPGMA and the model of binary Jaccard into a **(B)** PCA with group A representing rhizospheric soil and group B representing bulk soil; **(C)** clustering of samples using a heat map analysis.

Supplementary Figure 6 Taxonomic variation at the **(A)** phylum level and **(B)** order level.

Supplementary Figure 7 Clustering of the samples using flower or Venn diagrams.

Supplementary Figure 8 Taxonomic variation at the **(A)** phylum and **(B)** order level.
