# Supplementary material for: Permanent crop cover as a strategy for drought-resistant viticulture: insights on how rhizosphere metagenomics influences leaf-level -omics for an enhanced overall plant response
Source: Front Plant Sci. 2025 May 29;16:1543171. doi: 10.3389/fpls.2025.1543171 (PMC12158955; doi:10.3389/fpls.2025.1543171)
Supplement: Supplementary Figure 1 — Image of the implementation of the crop cover into the experiment; picture taken 1-week previous to apply the water treatment. [file DataSheet1.pdf]

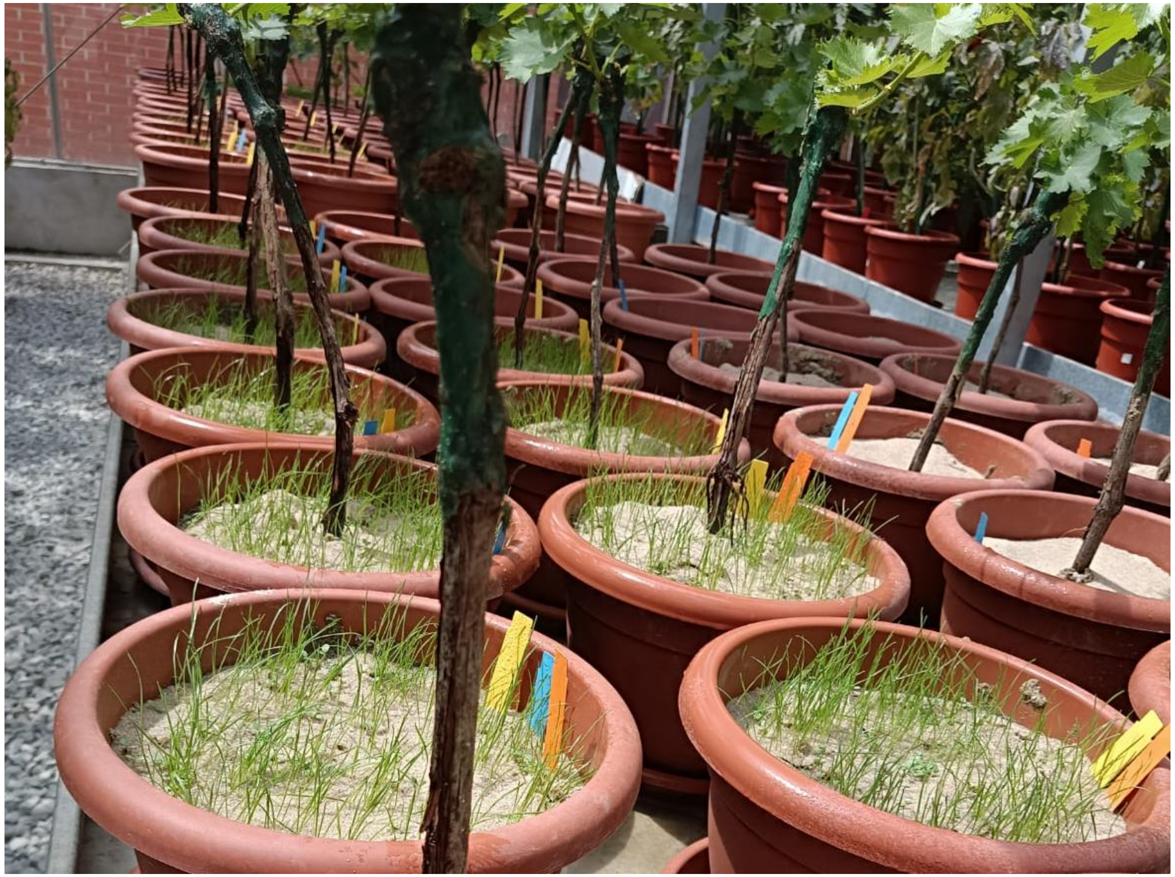

Supplemental Figure S1. Image of the implementation of the crop cover into the experiment; picture taken 1-week previous to apply the water treatment.

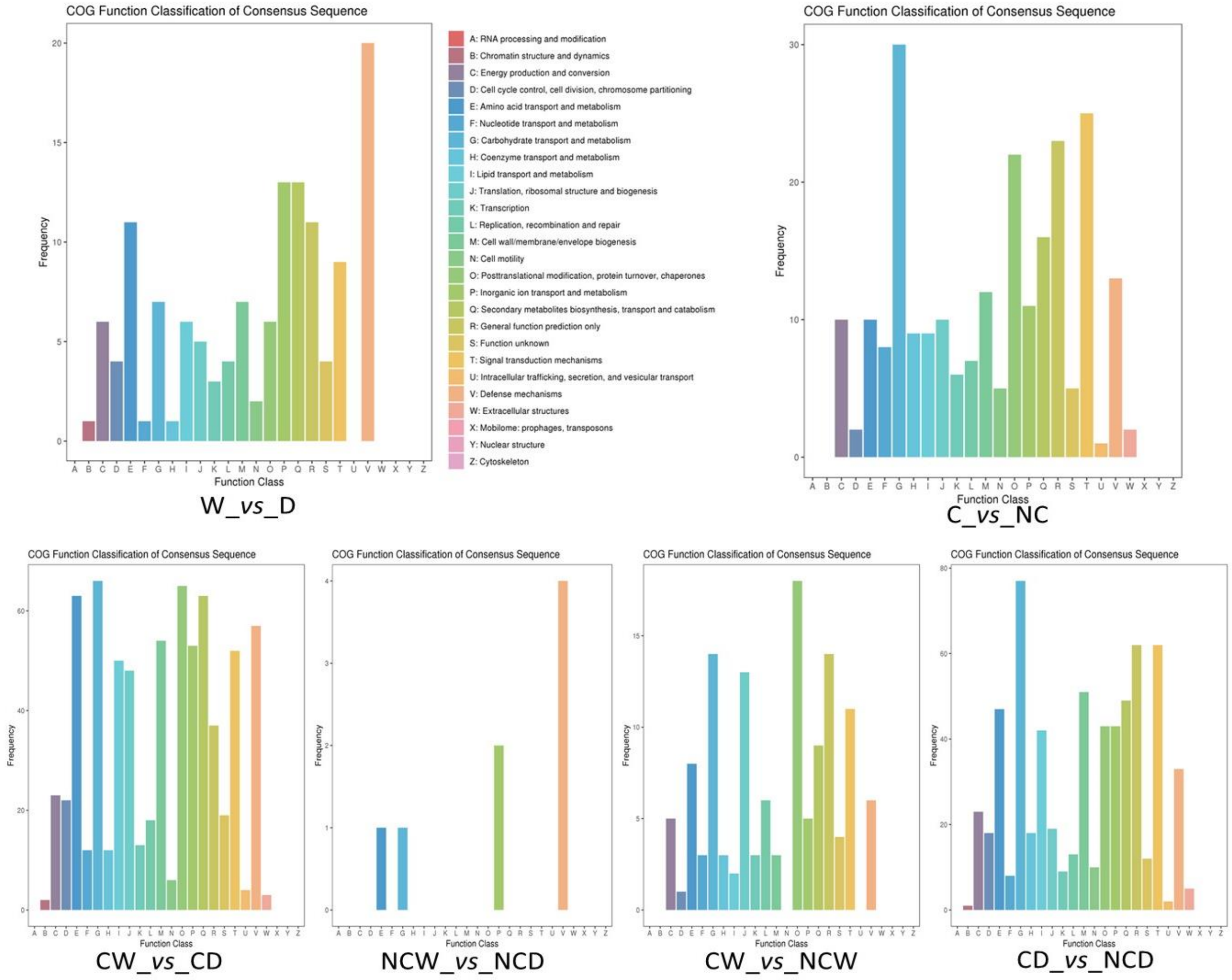

Supplemental Figure S2. Enrichment analysis based on cluster KOG

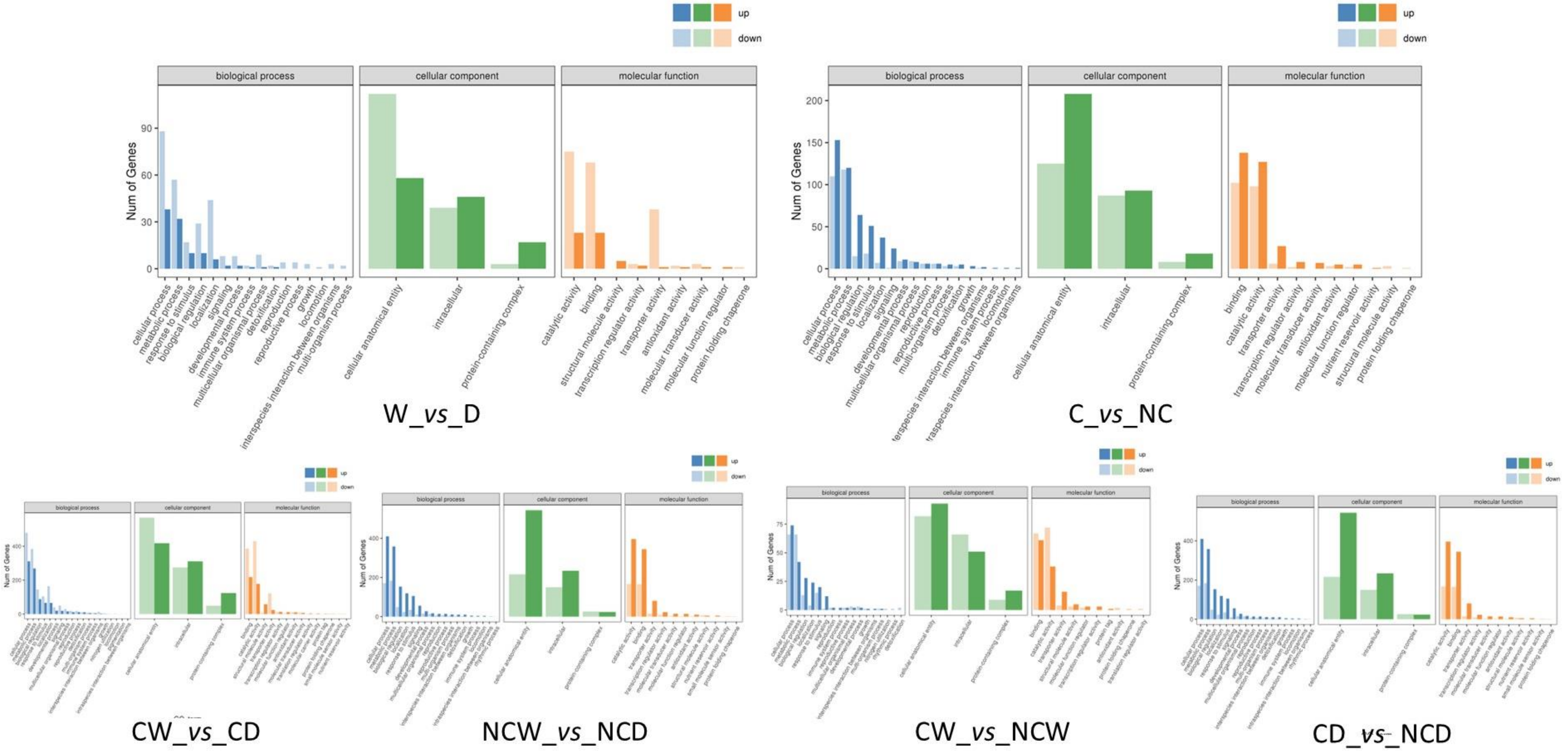

Supplemental Figure S3. Enrichment analysis based on cluster GO

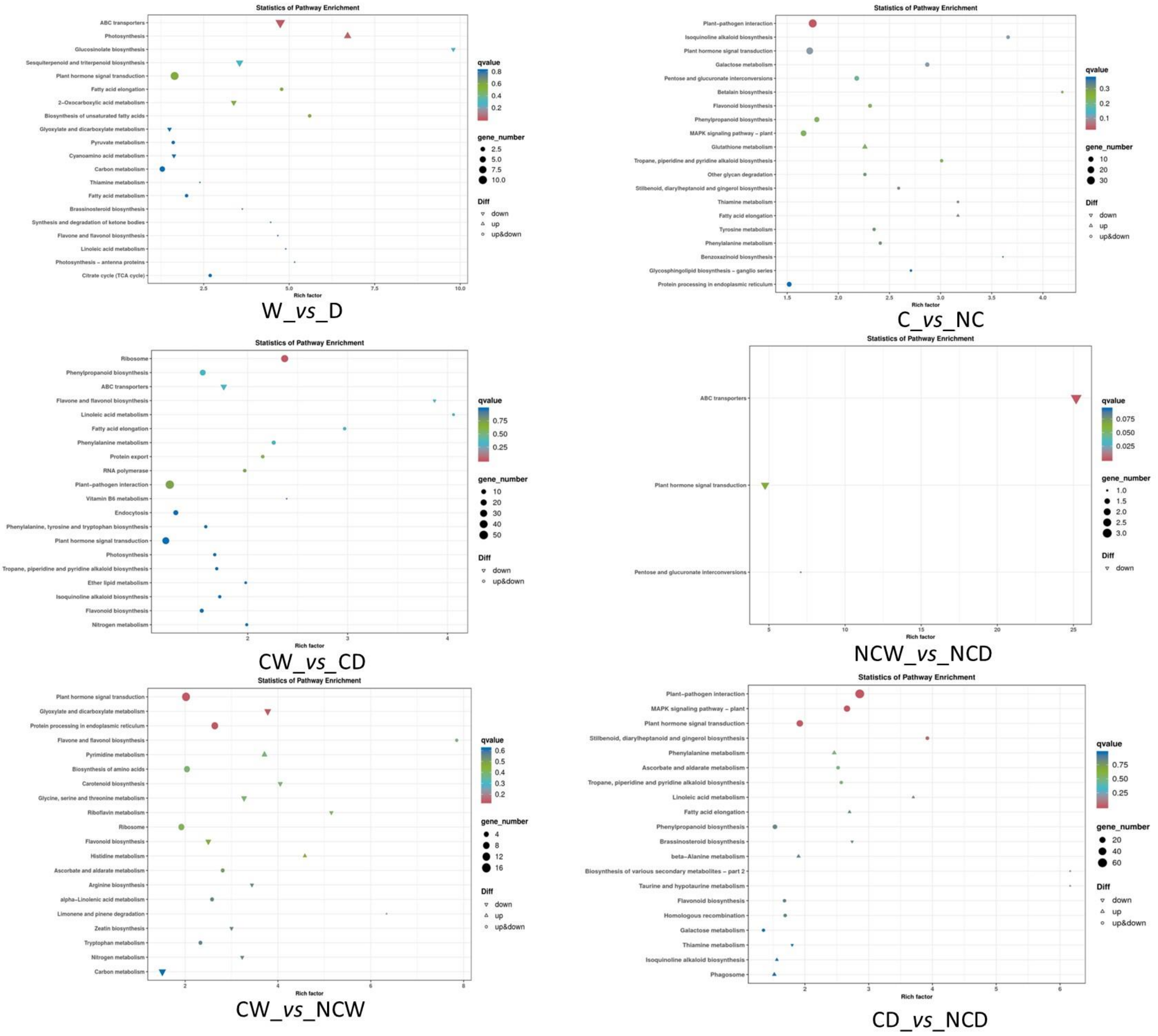

Supplemental Figure S4. Enrichment analysis based on cluster KOG.

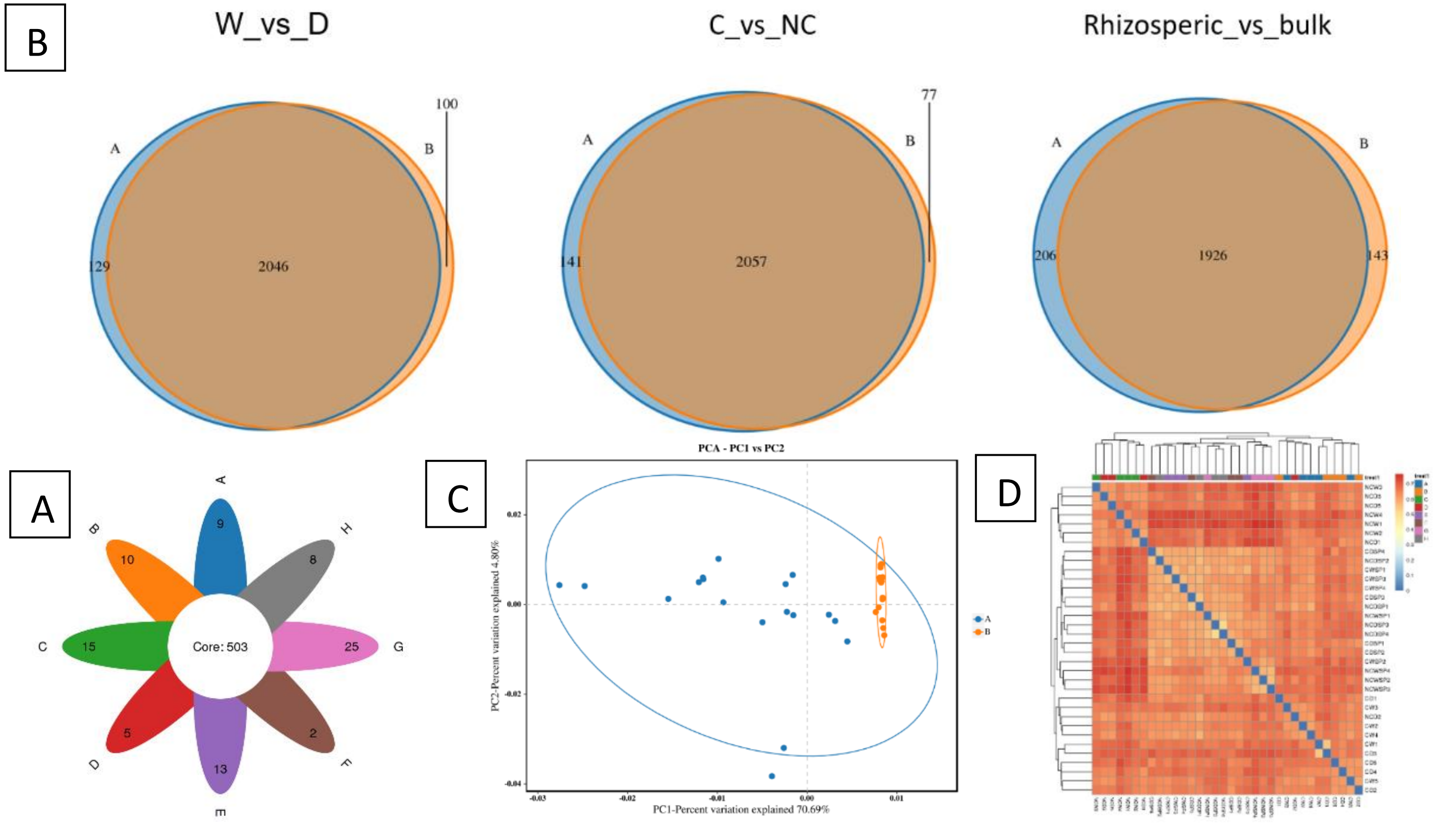

Supplemental Figure S5. Clustering of the samples A) using flower or Venn diagrams; and using UPGMA and the model of binary jaccard into a B) PCA with group A representing rhizospheric soil and group B representing bulk soil; C) clustering of samples using a heat map analysis.

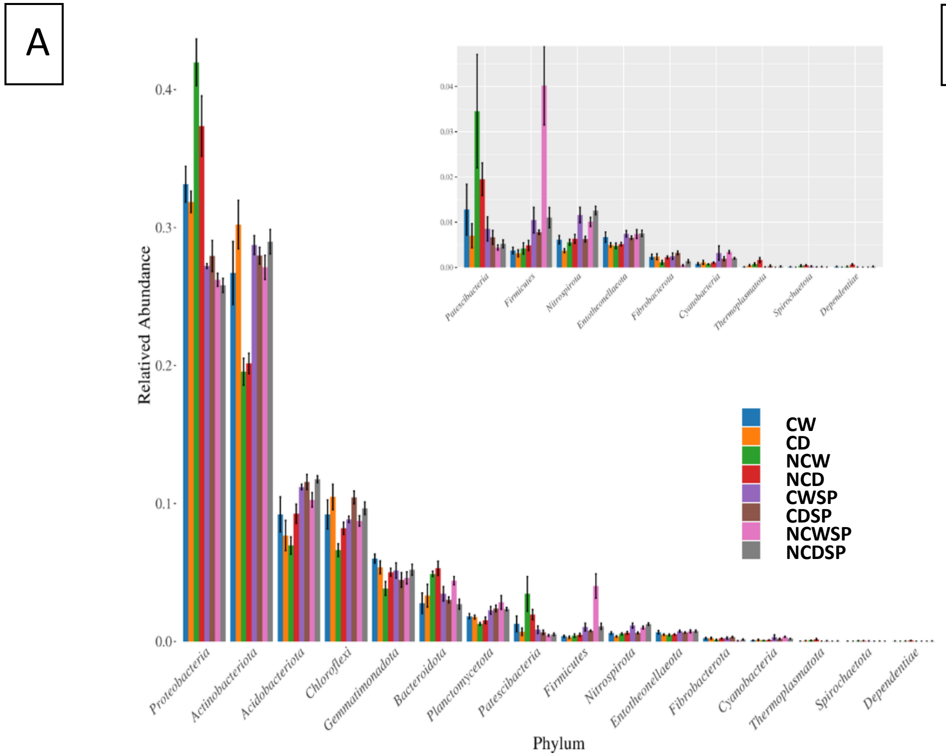

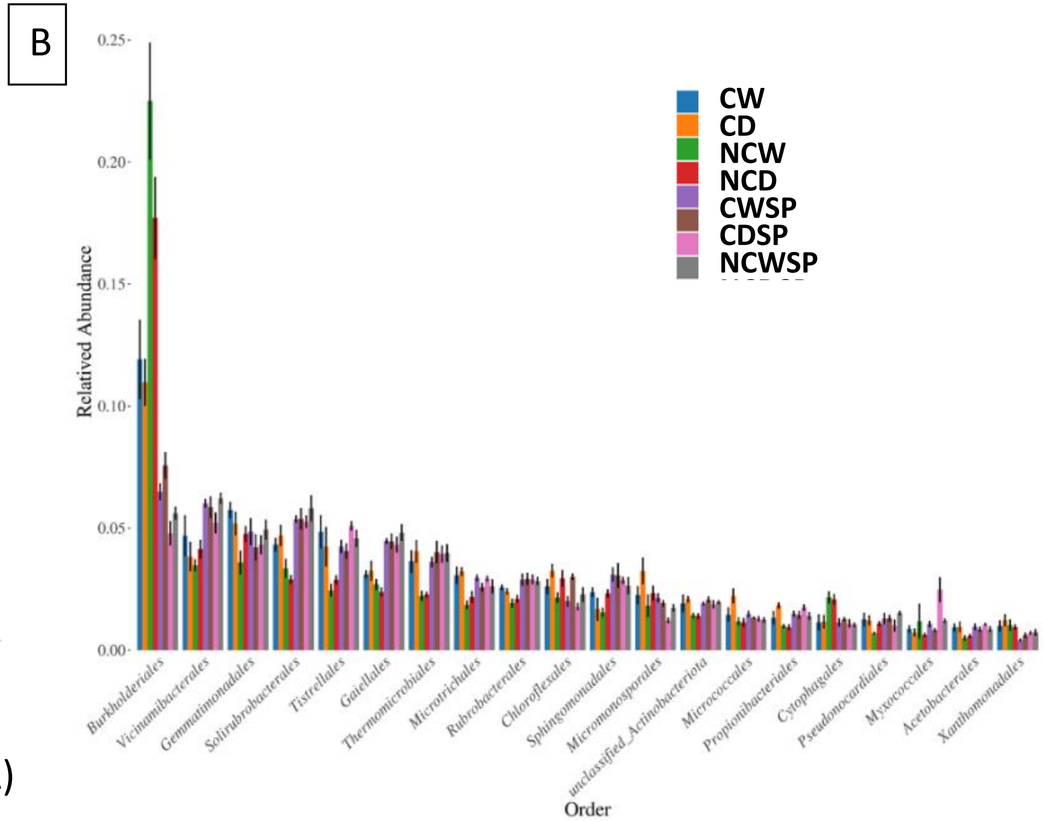

Supplemental Figure S6. Taxonomic variation at the A)

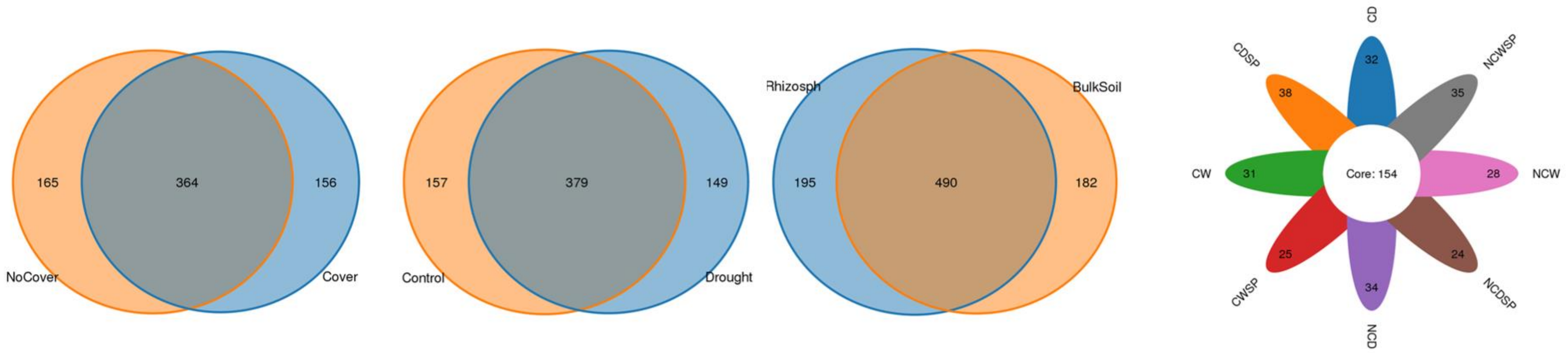

Supplemental Figure S7. Clustering of the samples using flower or Venn diagrams.

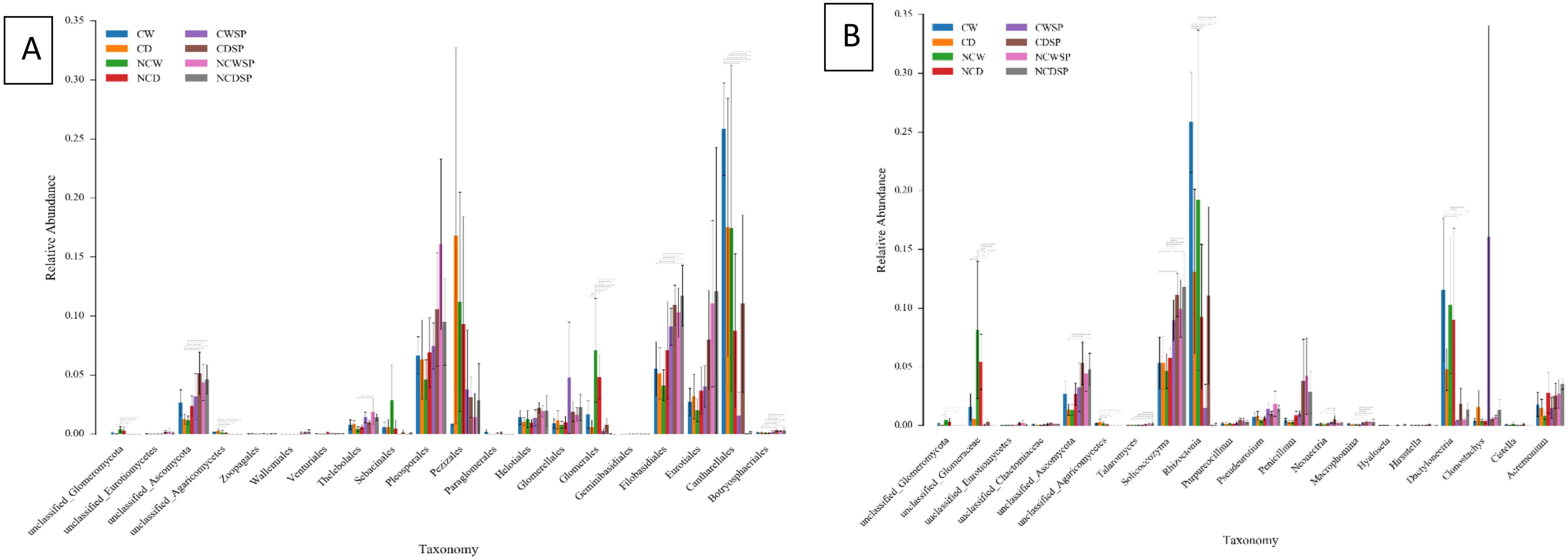

Supplemental Figure S8. Taxonomic variation at the A) phylum and B) order level.
